# Extraordinary Case of Retention of Urine

**Published:** 1869-02

**Authors:** J. H. Curtis

**Affiliations:** Kansas City, Mo.


### ARTICLE X.

### EXTRAORDINARY CASE OF RETENTION OF URINE.

BY J. H. CURTIS, M.D., Kansas City, Mo.

Kate H., 14 years of age, lives near Wyandott, Kansas; brought to the office September 3, 1868; has pale, sallow complexion; tongue pale, flabby, and heavily coated; pulse regular, and not too rapid; abdomen but very little enlarged, and without tympanitis, and without tenderness or pain. But complains of some pain, with a curious feeling in head; has never menstruated; bowels regular, but without any desire to micturate. There is a very considerable increase of size about the pelvic region, with a spreading of the hips like a woman in the last stage of pregnancy, but without any evidence of dropsical effusion either in the cavities or cellular tissue. Declares positively she has not voided a drop of urine for four weeks and four days; her statement is corroborated by her mother, who has been with her constantly, watching her closely when she would go out to stool, and asserts it has been absolutely impossible for her to have passed any urine without her knowing it; and for 32 days not one drop of water has passed from her as urine. Has been under treatment a number of weeks from three or four doctors in their neighborhood, but without any benefit; catheterism having been tried twice without any result.

I gave her immediately fl. ex. hydrangea, ℥iss., to be repeated every three hours, and to be followed in 1½ hour with a powder of calomel, grs. xx., to be repeated at the same intervals after each dose of the hydrangea, until the bowels should be well moved. In 14 hours after she had taken five doses of the fl. ex. and four powders, and before the bowels had moved, after suffering the most intense pain for an hour previous, she passed 3½ quarts of very clear, colorless urine; the bowels were well moved in an hour or two afterwards, and in six hours she again voided 4 quarts more of the pellucid-looking water, by which she was entirely relieved. In two weeks complete convalescence was established, by the use of pil. hydrarg. every third night, and the use of nit. potass. and soda bicarb. solution every three hours during the daytime.

Having never heard or read of such an extreme case of retained urine, I have thought it worth reporting; and the result of the treatment, so prompt and satisfactory, may benefit some of my medical brethren, if called to meet a similar case. The mother and daughter are both willing to make affidavit to the above statement.

